# Slow, progressive myopathy in neonatally treated patients with infantile-onset Pompe disease: a muscle magnetic resonance imaging study

**DOI:** 10.1186/s13023-016-0446-7

**Published:** 2016-05-17

**Authors:** Steven Shinn-Forng Peng, Wuh-Liang Hwu, Ni-Chung Lee, Fuu-Jen Tsai, Wen-Hui Tsai, Yin-Hsiu Chien

**Affiliations:** Department of Radiology, National Taiwan University Hospital, Taipei, Taiwan; Department of Medical Genetics, National Taiwan University Hospital, Taipei, Taiwan; Department of Pediatrics, National Taiwan University Hospital, Taipei, Taiwan; Department of Pediatrics, National Taiwan University College of Medicine, Taipei, Taiwan; Department of Pediatrics, College of Chinese Medicine, Taichung, Taiwan; Department of Pediatrics, Chi Mei Medical Center, Tainan, Taiwan

**Keywords:** Glycogen Storage Disease Type II, Magnetic Resonance Imaging, Neonatal Screening, Enzyme Replacement Therapy

## Abstract

**Background:**

Patients with infantile-onset Pompe disease (IOPD) can be identified through newborn screening, and the subsequent immediate initiation of enzyme replacement therapy significantly improves the prognosis of these patients. However, they still present residual muscle weakness. In the present study, we used longitudinal muscle magnetic resonance imaging (MRI) to determine whether this condition is progressive.

**Materials and methods:**

A cohort of classic IOPD patients who were diagnosed through newborn screening were treated with recombinant human acid α-glucosidase (rhGAA) and followed prospectively from birth. The trunk (and abdominal wall), pelvis and upper thighs were scanned for muscle MRI every 2–3 years. Seven groups of muscles were individually scored from 0 to 4 based on the extent of their involvement, and the sum was correlated to the clinical manifestations.

**Results:**

Twenty-four MRI scans from a total of 12 neonatally treated IOPD patients were analyzed in the present study. The median age at the time of MRI scanning was 4.2 years (13 days to 9 years). High intensity over the quadriceps on T2-weighted and short-tau inversion recovery images was observed in all scans and was followed by a decrease in muscle mass. Trunk muscle involvement was slower, except in one patient who exhibited progressive psoas atrophy. Among the 10 patients for whom follow-up scans were repeated more than 2 years after the first scan, four patients (40 %) showed increased myopathy severity.

**Conclusion:**

This prospective muscle MRI study provides evidence for the occurrence of slow, progressive muscle damage in neonatally treated IOPD patients during childhood. New treatment strategies are necessary to improve outcomes in these patients.

**Electronic supplementary material:**

The online version of this article (doi:10.1186/s13023-016-0446-7) contains supplementary material, which is available to authorized users.

## Background

Pompe disease is a lysosomal storage disorder in which a deficiency of acid α-glucosidase (GAA, EC 3.2.1.20) causes the intralysosomal accumulation of glycogen in all tissues, most notably in skeletal muscles [1]. Enzyme replacement therapy (ERT) with recombinant human GAA (rhGAA) [2, 3] is currently the only causal treatment available for patients with Pompe disease. However, muscle involvement does not respond well to ERT. It has been suggested that late treatment does not rescue damaged muscles, and glycogen clearance was observed in nearly all tissues from two patients who received early ERT and exhibited good clinical responses at 96 and 77.5 months after the start of ERT [4].

To achieve early diagnosis and treatment, we initiated a large-scale population newborn screening program for Pompe disease in 2005 [5]. This program successfully identified patients with classic infantile-onset Pompe disease (IOPD), but also patients with late-onset Pompe disease (LOPD). The benefits of ERT in classic IOPD patients who were identified through newborn screening and treated since birth have been demonstrated in both survival and ventilation-free survival [6]. However, pelvic girdle weakness gradually developed in these patients after two years of age and was associated with an increase in creatine kinase (CK) levels [6]. Elevations in CK levels have been associated with increased urinary glucose tetrasaccharide (Glcα1-6Glcα1-4Glcα1-4Glc, Glc4) excretion [7], indicating worsening glycogen accumulation in the muscles [8]. In neonatally treated IOPD patients, quadriceps biopsies obtained at 6 months after the onset of ERT revealed significant glycogen clearance [9, 10]; however, follow-up biopsies are rarely performed because the procedure is invasive. In addition, muscle biopsy provides only localized information. Recently, magnetic resonance imaging (MRI) has shown initial success in detecting abnormalities in multiple muscle groups that are frequently involved in late-onset Pompe disease [11], even before physical examination [12], and these measurements could serve as indicators of disease progression. In the present study, we employed MRI to monitor the progression of myopathy in a prospective study involving neonatally treated patients with classic IOPD.

## Methods

Patients were selected using a newborn screening program directed through the National Taiwan University Hospital Newborn Screening Center, and the clinical information has been previously reported [6]. Patients were numbered by previous studies that NBS indicates classic IOPD detected by screening, NBSL indicates LOPD detected by screening, and CLN indicates clinically diagnosed patients. NBS patients were treated with 20 mg/kg of rhGAA every 2 weeks starting from age 6–39 days, and the follow-up period was 28–90 months [6]. Due to persistent elevation of CK and slow improvement in motor development, inadequate muscle treatment efficacy was suspected; therefore, increasing the dosage and/or frequency was attempted at various ages. Patients with LOPD were also selected during the same period and four were included for comparison [13, 14]. All patients participated in a long-term prospective follow-up study for Pompe disease that was approved by the local ethics committee, and written informed consent was obtained from the parents.

All patients were followed since birth. One IOPD diagnosed by clinical symptoms (CLIN3) but not NBS was also included in the follow-up program and his data was also included. For the IOPD patients, muscle MRI of the trunk (including the abdominal wall), pelvis and upper thighs was performed approximately every 2 to 3 years. For classification, patient age at the time the MRI was performed was categorized as infancy (under 1 year), toddler (under 4 years), preschool (under 6 years), and child (over 7 years). MRI was performed using a 1.5-Tesla scanner (Signa HDXt; GE Medical Systems, Milwaukee, WI, U.S.A.) (*n* = 10) or a 3-T MRI system (Siemens Healthcare, Erlangen, Germany) (*n* = 2) with an 8-channel, phased-array torso. All uncooperative patients received chloral hydrate (prepared at National Taiwan University Hospital) per os at a dose of 0.5-0.7 mg/kg BW 10–45 min prior to MR examination. Fast spin-echo T2-weighted images (T2WIs) were collected using the following parameters: long TR, fast spin echo (TR/TE = 3067-8000/78-100 ms) with a flip angle of 90–150, echo-train length of 15–20, and acquisition in one axial plane. Spin-echo T1-weighted images (T1WIs) were obtained using the following parameters: short TR, fast spin echo (TR/TE = 540-767/9.8-20 ms) with a flip angle of 90, echo-train length of 3, and one acquisition in both axial planes. In addition, all patients also received short-tau inversion recovery (STIR) MRI examinations using the following parameters: long TR (TR/TE/TI = 3000-6317/30-90/150-200 ms) with a flip angle of 90–150, echo-train length of 10, and acquisition in one coronal plane. The imaging matrix was 256 x 192 or 256 pixels for the T2WIs, T1WIs and STIR images. The slice thickness for the T2WIs and T1WIs was 4–5 mm with an intersection gap of 1 mm. Total scanning time per patient was 30–40 min.

Abnormalities in the trunk, pelvic and upper thigh muscles were described first. To enable better comparisons, especially long-term comparisons, the severity of muscle involvement was scored for each muscle using a 4-point classification system: (1) normal appearance; (2) mild involvement (involvement of less than 50 % of the area); (3) moderate involvement (involvement of more than 50 % of the area; and (4) severe involvement, including fatty replacement or atrophy [15]. Seven muscles were scored, including the erector spinae, psoas, gluteal, rectus femoris (RF), vastus lateralis (VL), vastus intermedius (VI), and adductor magnus muscles. The severity scores were correlate with serum CK and Pompe-PEDI (Pediatric Evaluation of Disability Inventory specific for Pompe disease) mobility normative scores using Spearman’s correlation (SPSS software, version 17.0 for Windows). The Pompe-PEDI [16] was used to assess the motor capability required for participating in daily locomotion and transfer tasks, and the normative standard scores (adjusted for the child’s chronological age) of the mobility domain of the Pompe-PEDI Functional Skills Scales were used. Higher scores reflect a superior capability. The values below limit of qualification of Pompe-PEDI mobility normative scores (i.e. 10) were shown as 1/2 (i.e. 5) for this correlation analysis.

## Results

### Description of patients

Twenty-four MRI scans were obtained from a total of 12 patients in the present study; 2 patients received one MRI scan, 8 patients received two MRI scans, and 2 patients received 3 MRI scans (Table 1). These patients have been previously described [6, 17]. All of the patients tested positive for cross-reactive immune material (CRIM), and none of the patients exhibited sustained high titers of anti-rhGAA. The median age at the time of MRI scanning was 4.2 years, with the first MRI scan performed at 13 days to 6 years of age, and the latest performed at 7 months to 9 years of age. Five MRIs were performed in the infancy age group (from 4 out of the total 12 patients; 33 %), while 8 (67 % of patients) were performed in the toddler, 7 (87.5 %) in the preschool, and 4 (67 %) in the child age groups. In this study, the median age of the tested patients was 7.6 years (2.8-9.2), and all individuals walked independently. The median CK level at the time of MRI was 1025 U/L (range 198–2500). Four LOPD patients who were identified through this newborn screening program were included for comparison; three patients presenting with persistent CK elevation or frequent falls were undergoing ERT.

**Table 1:** Clinical, genetic, and muscle MRI findings in 12 patients with infantile-onset Pompe disease and 4 patients with late-onset Pompe disease

No^a^	Age of ERT start	Current age (yr)	GAA gene		Each MRI scan			
			Mutation 1	Mutation 2	Age (yr)	MRI Score	CK (U/L)	Mobility scores
NBS2	26 D	9.2	c.1935C > A^b^	c.1411_1414del^c^	4.9	17	595	20.2
					8.1	24	2500	<10
NBS3	29 D	9.1	c.1935C > A	c.2842insT	4.0	19	2135	18.46
					5.8	23	2444	<10
					8.0	25	1957	<10
NBS4	17 D	8.5	c.1935C > A	c.784G > A	6.3	22	1642	12.12
					8.4	22	1674	22.51
NBS5	34 D	8.0	c.1935C > A	c.1935C > A	2.9	15	1238	33.33
					4.9	21	1663	26.05
NBS6	12 D	7.6	c.1935C > A	c.[1062C > G; 1286A > G]	2.5	18	735	35.89
					6.1	18	596	21.88
NBS8	14 D	6.2	c.1935C > A	c.1197_1208del	6.2	21	1156	4.64
NBS9	39 D	5.9	c.1935C > A	c.2040 + 1G > T	0.6	12	198	32.90
					4.4	9	1030	30.83
NBS10	6 D	4.5	c.1935C > A	c.1935C > A	1.0	14	733	44.05
					3.0	12	1020	58.89
NBS11	14 D	4.4	c.1935C > A	c.1935C > A	3.0	16	941	30.06
NBS12	16 D	4.1	c.1935C > A	c.1197_1208del	1.3	18	246	24.44
					3.1	22	673	24.53
NBS14	10 D	2.8	c.1935C > A	c.1935C > A	0.0	-	637	-
					0.6	16	344	-
					3.1	15	760	-
CLN3	3 M	9.0	c.1935C > A	c.1411_1414del^c^	6.5	20	1904	26.87
					9	19	1565	24.40
NBSL2	3 Y	8.4	c.2238G > C	c.2662G > T	1.5	11	267	52.19
					7.3	11	267	38.74
NBSL6	3.5 Y	7.7	c.1935C > A	c.[752C > T; 761C > T]	2.7	7	113	39.85
					5.8	7	222	37.85
NBSL12	-	6.4	c.1935C > A	c.[752C > T; 761C > T]	3.5	7	87	60.99
NBSL16	4 M	4.6	c.1935C > A	c.1634C > T	0.4	7	861	-
					3.3	7	173	53.03

*D* days, *M* months, *Y* years; MRI score: MRI severity scores from seven groups of muscles; Mobility scores: Pompe PEDI (Pediatric Evaluation of Disability Inventory specific for Pompe disease) mobility normative scores

^a^Patients were numbered by previous studies that NBS indicates classic IOPD detected by screening, NBSL indicates LOPD detected by screening, and CLN indicates clinically diagnosed patients

^b^This mutation is always associated with the c.1726G > A mutation

^c^This mutation is associated with the c.[752C > T; 761C > T] mutation

### MRI changes during infancy

**Fig. 1:**
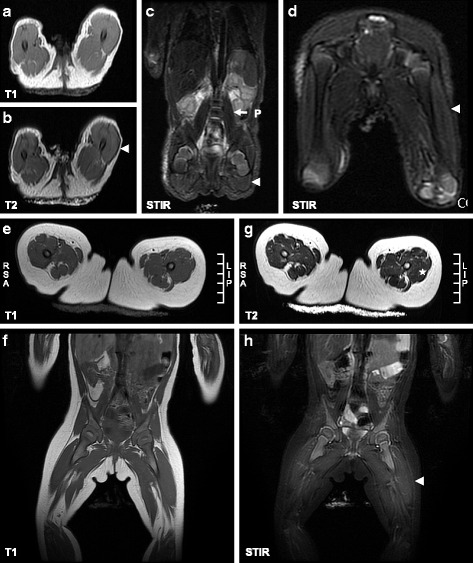
Earliest muscle MRI changes in newborn patients with IOPD. **a**-**d** At 13 days of age, no significant signal changes in the thigh were revealed on T1WIs (**a**) obtained from NBS14, with equivalent findings on T2WIs (**b**). However, significant signal changes were observed on STIR images (**c**, **d**), which revealed hyperintensity, particularly in the vastus lateralis (arrowhead). **e**-**h** At 7 months of age, hyperintensity was noted in the anterior region of the thigh muscles on T2WIs (G, asterisk) and STIR images (**h**, arrowhead) but not on T1WIs (**e**, **f**)

All patients with IOPD presented with CK elevation and muscle glycogen accumulation at the time of diagnosis. MRI scans were obtained from one patient at 13 days and 7 months of age (Fig. 1). At 13 days of age, although her muscle biopsy revealed intracytoplasmic vacuoles in more than 90 % of the myocytes, there were no signal changes on the T1WIs. Nevertheless, consistent hyperintensity was detected in the thigh muscles on STIR images and T2WIs, and this effect was more prominent in the VL. After ERT for 7 months, a repeat MRI revealed hyperintensity on T2WIs and STIR images but not on T1WIs.

### T1WI-negative changes during early childhood

**Fig. 2:**
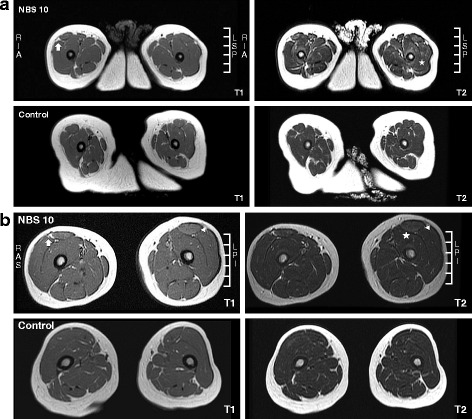
Muscle MRI in neonatally treated infants and toddlers with IOPD. **a** T1WIs (left panel) and T2WIs (right panel) of thigh muscle MRI scans obtained at 1 year of age from NBS10 (upper panel) and an age- and sex-matched control (lower panel) were compared. NBS10 presented hyperintensity at the bilateral quadriceps, particularly the vastus intermedius, on T2WIs (asterisk) but not on T1WIs. In addition, increased fat in the intramuscular fascia (between the vastus lateralis and rectus femoris) was also observed (arrow). **b** T1WIs (left panel) and T2WIs (right panel) of thigh muscle MRI scans obtained at 3 years of age from NBS10 (upper panel) and an age- and sex-matched control (middle panel) were compared. NBS10 presented hyperintensity at the bilateral vastus intermedius and less hyperintensity at the vastus lateralis on T2WIs (asterisk) but not on T1WIs, showing a significant decrease compared with previous images obtained at 1 year of age. In addition, hyperintensity at the bilateral rectus femoris (triangles) was clearly evident on both T1WIs and T2WIs. Increased fat in the intramuscular fascia (between the vastus lateralis and rectus femoris) was also observed but had not progress (arrow)

The hyperintensity observed in T2WIs and STIR images persisted in older patients (Fig. 2). At one year of age, NBS10 presented hyperintensity over the anterior region of the thigh, particularly in the VI, on T2WIs but not on T1WIs. At age 3, the lesions on VI were decrease. Those on the VL appeared only on STIR images, and those on the RF were detected on both T1WIs and T2WIs. At 1 year of age, an increase in intramuscular fat was also observed, suggesting a decrease in muscle mass.

### T1WI-positive changes in childhood

**Fig. 3:**
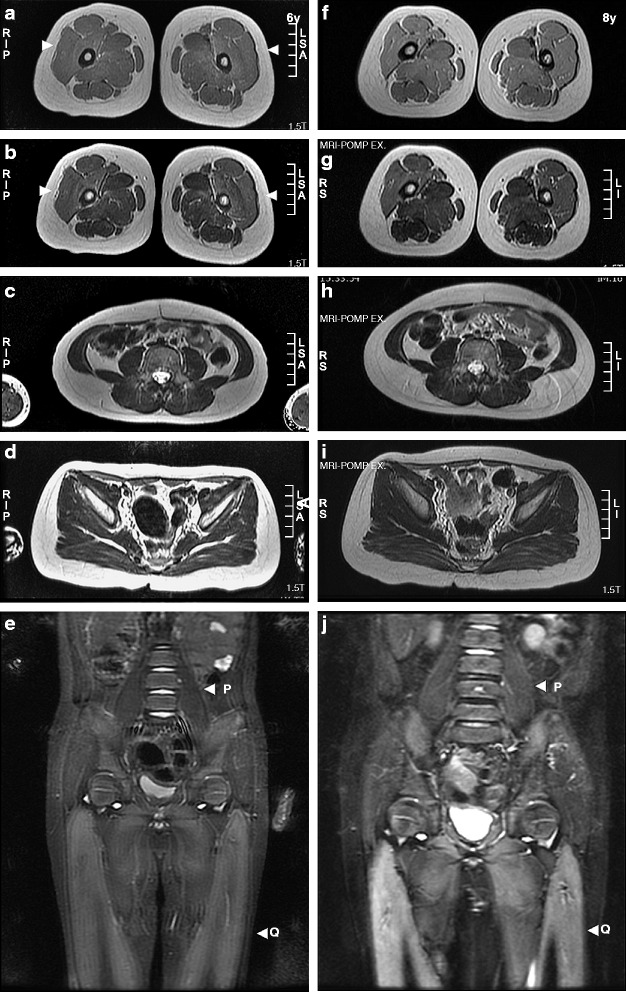
Sequential MRI studies in neonatally treated patients with IOPD. (**a**) Imaging of NBS4 was performed at 6 (left panel) and 8 years (right panel) of age. Changes were most severe in the thigh muscles (arrowheads), followed by the trunk and pelvic muscles. STIR revealed the rapid progression of hyperintensity over the quadriceps muscles. (*a*, *f*) Thigh T1WIs, (*b*, *g*) thigh T2WIs, (*c*, *h*) trunk T2WIs, (*d*, *i*) pelvic T2WIs, and (*e*, *j*) coronal STIR images. Q: quadriceps muscles; P: psoas muscles. (**b**) Similar thigh T2WI changes in patients examined at 5 (*a*–*c*) and 8 years (*d*–*f*) of age. T2WI signal changes and muscle atrophy were prominent, particularly over the quadriceps muscles in the IOPD patients but not in the LOPD patients who were administered rhGAA therapy: (*a*, *d*) NBS2, (*b*, *e*) NBS3, and (*c*, *f*) NBSL2. (**c**) Variable degrees of thigh T2WI changes in patients examined at 3 (*a*–*c*) and 5 years (*d*–*f*) of age. T2 signal changes over the quadriceps muscles and increased intramuscular fat were prominent the IOPD patients but not in the LOPD patients who were administered rhGAA therapy: (*a*, *d*) NBS5, (*b*, *e*) NBS6, and (*c*, *f*) NBSL6

The older patients presented more extensive changes. In addition to hyperintensity in T1WIs and T2WIs over the anterior region of the thigh, other thigh muscles were also affected, such as the adductor magnus and semimembranosus muscles. In addition, the muscle mass was decreased, as shown using small quadriceps calibers, and the subcutaneous fat was significantly increased (Fig. 3). T1WI hyperintensity was also evident as early as 3 years of age in NBS12 (Additional file 1: Figure S1). All patients presented thin rectus abdominis muscles, with some hyperintensity in the external and internal oblique muscles and the transversus abdominis muscle. The trunk (psoas, iliocostalis, longissimus and multifidus muscles) and gluteal muscles were more gradually affected than the thigh muscles (Additional file 2: Figure S2).

Among the examined muscles, the progression of the psoas muscles varied (Additional file 3: Figure S3). For example, psoas muscle atrophy was observed upon the first examination of NBS3, who could barely stand straight, likely because of prior fat infiltration in the psoas muscles, as demonstrated in NBS2 at 8 years of age and in NBS12 at 3 years of age.

### Progression of myopathy

**Fig. 4:**
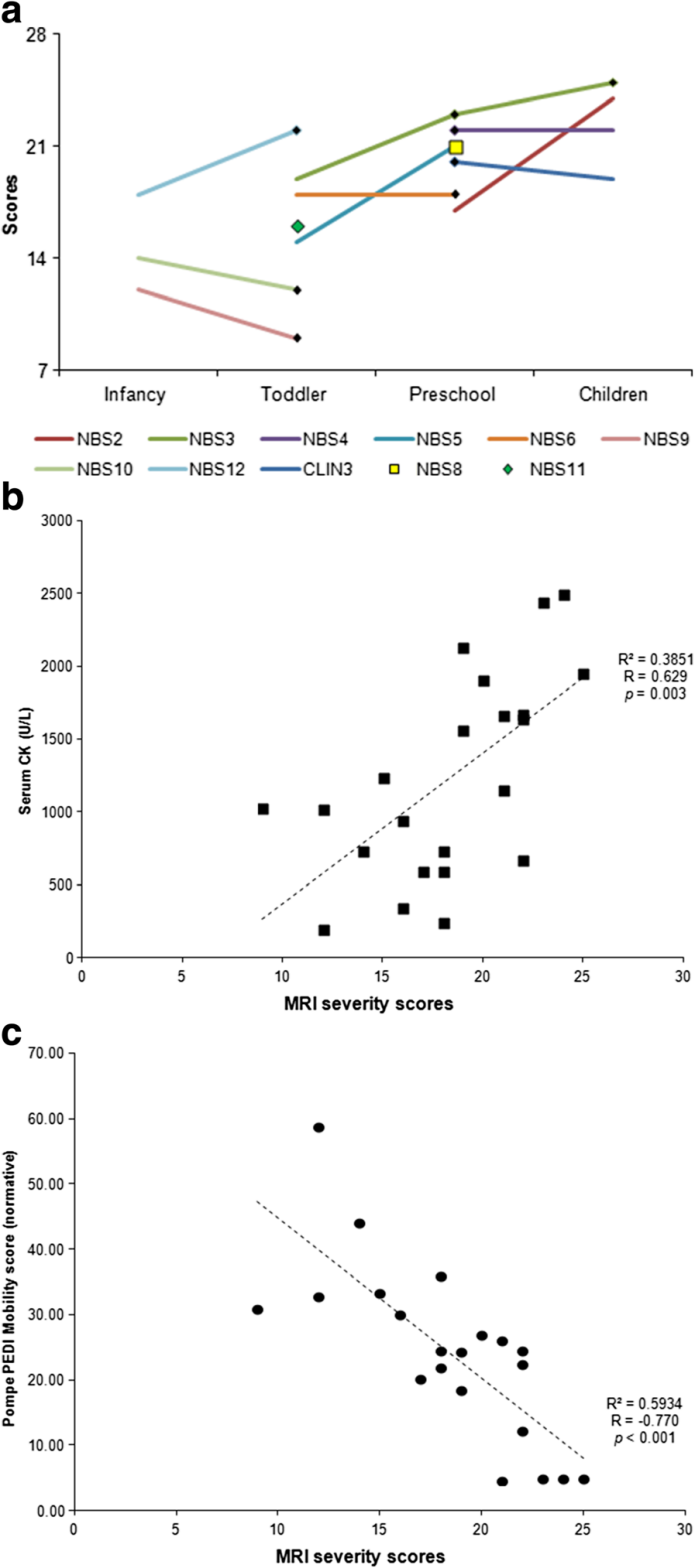
Correlations of trunk, pelvic, and thigh muscle MRI involvement severity scores. The severity score was positively correlated with serum CK levels (**a**) and negatively correlated with mobility function (**b**)

We therefore scored the severity of the commonly affected muscles, including the erector spinae, psoas, gluteal, RF, VL, VI, and adductor magnus muscles (Table 1). Point totals that exceeded 7 were considered abnormal. Among the 10 patients for whom at least one follow-up scan was obtained at least 2 years after the first scan, the scores of 4 patients (40 %) showed deterioration with age, of 2 patients (20 %) showed improvement with age, and of 4 patients (40 %) remained stable, although NBS4’s images showed more extensive changes (Fig.3a and Additional file 4: Figure S4). The two patients with decreased scores were NBS9 and NBS10. NBS10 was administered treatment at 6 days of age, and NBS9, who was born prematurely, was administered treatment at 35 weeks post-conception. These two patients also had higher rhGAA dosages at the time of the second scans. In addition, some of the other patients were receiving higher rhGAA dosages at the time of the second scan or had received an increase in dosage between the 2 scans, but we could not correlate the potential benefits of a higher rhGAA dosage with the MRI changes observed in our study because the follow-up protocol was not designed to evaluate the effects of dose changes. The severity scores were positively correlated with serum CK levels (R = 0.629, *p* = 0.002) (Fig. 4a) and negatively correlated with Pompe-PEDI mobility normative scores (R = −0.770, *p* < 0.001) (Fig. 4b).

## Discussion

### Summary of findings

To the best of our knowledge, detailed muscle MRI studies have not been previously reported for IOPD. The only report described pre-ERT findings in 6 IOPD patients who showed no signal changes on T1WIs and T2WIs [18, 19]. Because we conducted prospective monitoring of neonatally treated IOPD patients, the present study represents the first report of the sequence of selective muscle involvement, which featured early reversible or irreversible involvement of the quadriceps that was followed by more extensive changes within the thigh. However, all scans showed T2WI hyperintensity lesions over the thigh. The gluteal and trunk muscles, except the psoas muscle in NBS3, showed less severe and slower involvement. The progressive atrophy of the thigh muscles, as suggested by decreased muscle mass and separation of the muscles, was obvious when the patients were examined at 5–6 years of age. Previous observations of impairments in muscle mobility, as evaluated using the Pompe-PEDI at 4 years of age [6], suggest that the MRI findings obtained in the present study are relevant.

### Patterns of thigh involvement

The results obtained for neonatally treated IOPD patients revealed unique patterns of quadriceps involvement. In previous studies of juvenile- and adult-onset patients, the first MRI changes involved the posterior thigh and trunk muscles [12, 20, 21]. The rectus femoris is typically the least involved muscle [11] in juvenile and adult patients. However, in the present study and in childhood-onset patients [22], the involvement of trunk muscles (except the psoas muscle) appeared at a later age. The different patterns of involvement between early-treated IOPD and LOPD patients might reflect the variable efficacy of rhGAA in muscles at different developmental stages. In addition, the composition of muscle fiber types differs between children and adults, particularly the distribution and abundance of type 2b fibers [23, 24], which are less responsive to rhGAA [25].

### T2WI high intensity

The meaning of the high intensity observed on T2WIs in the present study is intriguing. High intensity on T2WIs might suggest fatty infiltration and edema/inflammation, but STIR suppresses fat signals and reveals only inflammation or edema [26]. Therefore, we suspected that the T2WI-positive process in the examined patients reflected edema or inflammation. In Duchenne muscular dystrophy (DMD), hyperintensity on T2WIs has been associated with muscle injury, repair, and inflammation [27]. Because obvious inflammation or necrosis is not commonly observed in muscle biopsied from patients with Pompe disease, the hyperintensity on T2WIs likely reflects edema. Consistent with this hypothesis, a recent study of muscle water content that was performed using quantitative nuclear MRI in adult Pompe patients demonstrated that 32 % of all muscles exhibited an increase in water content and that fatty degenerative changes took place faster in these muscles [28]. The increased water content might reflect cytosolic glycogen release from lysosomes, as glycogen storage disease type V patients similarly presented a slight increase in the intensity of muscle on T2WIs [29, 30] without fat replacement. Another possible explanation for the T2WI hyperintensity is denervation, which was described in a recent publication regarding a less obvious T2 high intensity in Charcot-Marie-Tooth disease 1A [31]. However, the T2WI intensity changes in the thigh were minimal compared to the changes in myopathy (inclusion body myositis).

In the current study, the early-onset STIR and T2WI hyperintensity were associated with normal T1WI intensity (T1WI-negative), which could be reversible. In contrast, T2WI and STIR hyperintensity in the older patients was associated with T1WI hyperintensity (T1WI-positive) that was followed by a significant decrease in muscle mass without obvious fatty replacement; this pattern differs from that observed in juvenile- and adult-onset Pompe disease patients. Differences in muscle-remolding ability between toddlers and children might explain the lack of fatty replacement before muscle atrophy observed in the patients examined in the present study.

### Implications for management

Here, we demonstrated by MRI studies a slow progressive myopathy in neonatally treated IOPD patients who continued to receive rhGAA at labeled (20 mg/kg every 2 weeks) or double dosages (40 mg/kg every other week or 20 mg/kg/qw). Only 2 patients, who also exhibited the earliest treatment initiation times, showed decreased muscle involvement at the last evaluation at 4 years of age when on higher dosages. Previous studies in adults have suggested a significant quantitative increase in muscle bulk in the lower limbs [32] after only 6 months of rhGAA treatment, but this increase was primarily observed in the spared muscles. Because muscle involvement in IOPD is more extensive than that in LOPD, treatment should be initiated prior to the evolution of irreversible muscle changes. We observed reversible T2WI changes prior to 3 years of age. Although further clarification such as correlation with muscle biopsy or EMG findings is needed, this finding suggests that earlier and more potent treatment might partially rescue those muscles. A recent study using even higher dose of rhGAA than our current usage suggests improvement in survival and motor function outcomes in IOPD patients [33], supporting our speculation.

### Limitations of this study

This prospective, long-term follow-up cohort study was conducted over a long period of time. Therefore, the methods used to obtain the MRI scans could have varied slightly, and different scanners were used. Thus, we used the relative intensity to visualize hyperintensity signals on MRIs. Second, because of time constraints and anesthesia-related risks, we only scanned the trunk/pelvis/thighs under sedation. This method produced some artifacts on the images because the patients were not completely anesthetized. However, we did not observe arrhythmia or other complications during this procedure. Third, we used severity scores and not quantitative measurements for the affected muscles. In the present study, we aimed to describe the pattern of involvement over time; therefore, the sensitivity of the severity scores might not be a critical factor. Moreover, we followed a small number of cases and acquired a low number of images. Thus, a longer follow-up with more patients will be necessary to confirm the observations reported here.

## Conclusions

The results of the present study showed that muscle MRI provides a non-invasive method to monitor muscle damage in IOPD patients. Without persistent antibody interference and neonatal initiation of ERT, the muscles remain damaged and present with edema. These symptoms are followed by slowly progressing muscle atrophy and fatty infiltration and the eventual loss of muscle function. Using a combination of MRI techniques, muscle involvement can now be characterized in more detail. Future longitudinal studies that complete serial examinations of muscles in IOPD patients would be valuable to extend the findings of the present study and provide further information regarding the treatment of IOPD.

### Ethics, consent and permissions

All procedures followed were in accordance with the ethical standards of the responsible committee on human experimentation (institutional and national) and with the Declaration of Helsinki of 1975, as revised in 2000. The Institutional Review Board approved this study, and written informed consent was obtained from the parents of all patients who were included in the study.

## Additional files

Additional file 1: Figure S1. Additional MRI findings in young patients. At age 3, NBS12 (IOPD), NBSL16 (LOPD under ERT for 2.6 years) and NBSL12 (LOPD without ERT) were compared. T1WIs (asterisk), T2WIs (asterisk) and STIR images (arrow) revealed significant hyperintensity in the vastus intermedius and vastus lateralis in NBS12 but not in NBSL16. In NBSL12, slightly increased signals were observed in STIR images but not T1WIs or T2WIs, but the quadriceps images showed increased intramuscular fat, indicating less muscle mass. (BMP 3062 kb) Additional file 2: Figure S2. Abdominal muscle involvement. All IOPD patients (A-E) presented with thin rectus abdominis muscles (triangles) and variable degrees of involvement of lateral muscles (transversus abdominis and external and internal oblique muscles) compared with a non-IOPD patient (F): (A) NBS3 at age 6 also demonstrated the atrophy of psoas muscles (arrow); (B) NBS5 at age 5; (C) NBS6 at age 6; (D) NBS8 at age 6; (E) NBS9 at age 4; and (F) NBSL6 at age 5. (BMP 730 kb) Additional file 3: Figure S3. Early-onset psoas muscle atrophy. MRI studies of patients NBS3 (A-D), NBS2 (E-G), and NBS12 (H-K) revealed variable signal changes and psoas muscle atrophy: Q: quadriceps and P: psoas. (BMP 1701 kb)

Additional file 4: Figure S4. Longitudinal follow-up of trunk, pelvic, and thigh muscle MRI involvement severity scores. For each muscle, involvement on T2WIs was scored from 1 (normal) to 4 (atrophy or total fat replacement). Seven muscles were scored, and the sum was plotted. Two of the 10 patients demonstrated improvement in muscle involvement. Black filled diamonds indicate the time under which the patients received a greater than labeled rhGAA dosage. NBS8 and NBS11 were receiving a higher dosage at the indicated time.

## Abbreviations

CK: creatine kinase
CRIM: cross-reactive immune material
DMD: Duchenne muscular dystrophy
ERT: enzyme replacement therapy
GAA: acid α-glucosidase
Glc4: Glcα1-6Glcα1-4Glcα1-4Glc
IOPD: infantile-onset Pompe disease
LOPD: late-onset Pompe disease
MRI: magnetic resonance imaging
Pompe-PEDI: Pediatric Evaluation of Disability Inventory specific for Pompe disease
RF: rectus femoris
rhGAA: recombinant human acid α-glucosidase
STIR: short-tau inversion recovery
T1WI: T1-weighted image
T2WI: T2-weighted image
VI: vastus intermedius
VL: vastus lateralis
