# Supplementary figures and images for: Slow, progressive myopathy in neonatally treated patients with infantile-onset Pompe disease: a muscle magnetic resonance imaging study
Source: Orphanet J Rare Dis. 2016 May 17;11:63. doi: 10.1186/s13023-016-0446-7 (PMC4869381; doi:10.1186/s13023-016-0446-7)

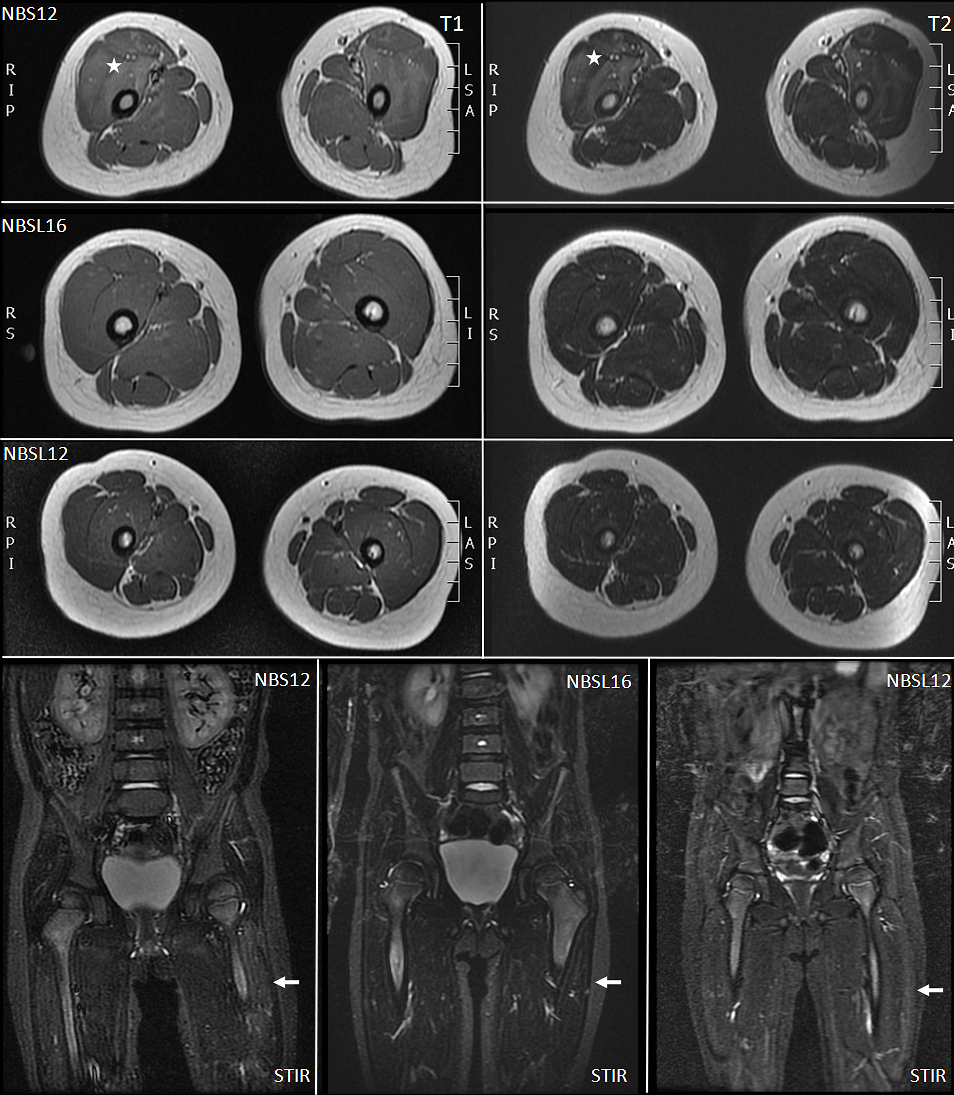

Supplement: Additional file 1: Figure S1. — Additional MRI findings in young patients. At age 3, NBS12 (IOPD), NBSL16 (LOPD under ERT for 2.6 years) and NBSL12 (LOPD without ERT) were compared. T1WIs (asterisk), T2WIs (asterisk) and STIR images (arrow) revealed significant hyperintensity in the vastus intermedius and vastus lateralis in NBS12 but not in NBSL16. In NBSL12, slightly increased signals were observed in STIR images but not T1WIs or T2WIs, but the quadriceps images showed increased intramuscular fat, indicating less muscle mass. (BMP 3062 kb) [file 13023_2016_446_MOESM1_ESM.bmp]

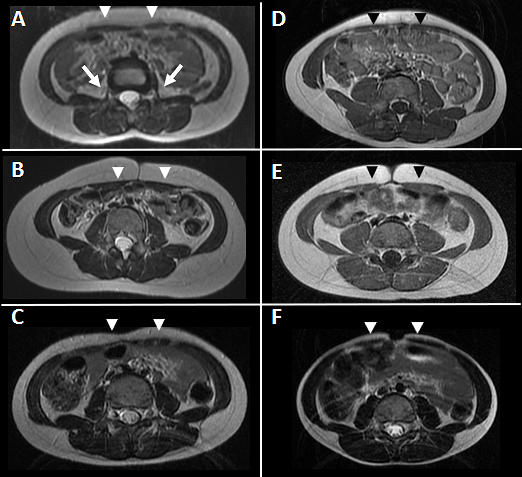

Supplement: Additional file 2: Figure S2. — Abdominal muscle involvement. All IOPD patients (A-E) presented with thin rectus abdominis muscles (triangles) and variable degrees of involvement of lateral muscles (transversus abdominis and external and internal oblique muscles) compared with a non-IOPD patient (F): (A) NBS3 at age 6 also demonstrated the atrophy of psoas muscles (arrow); (B) NBS5 at age 5; (C) NBS6 at age 6; (D) NBS8 at age 6; (E) NBS9 at age 4; and (F) NBSL6 at age 5. (BMP 730 kb) [file 13023_2016_446_MOESM2_ESM.bmp]

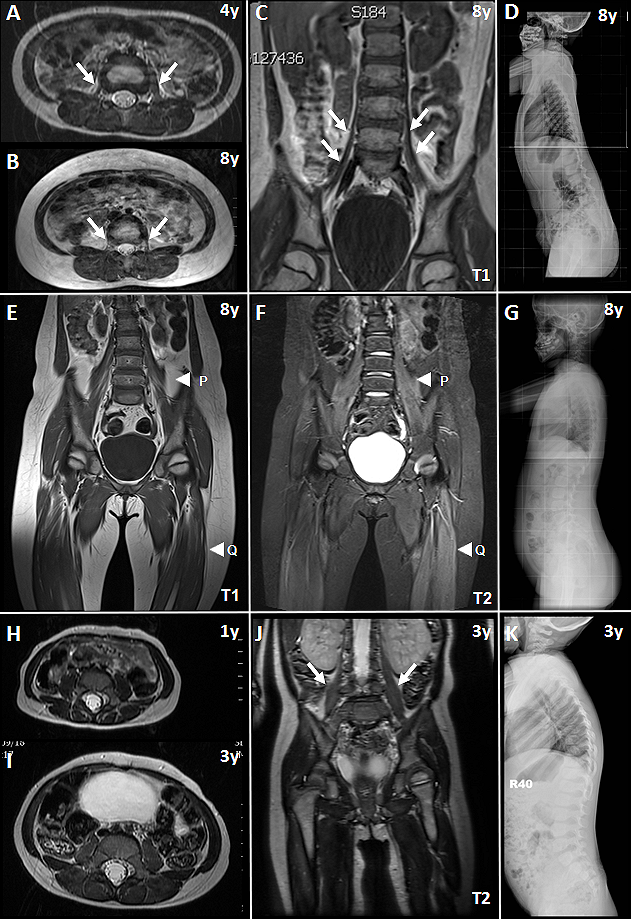

Supplement: Additional file 3: Figure S3. — Early-onset psoas muscle atrophy. MRI studies of patients NBS3 (A-D), NBS2 (E-G), and NBS12 (H-K) revealed variable signal changes and psoas muscle atrophy: Q: quadriceps and P: psoas. (BMP 1701 kb) [file 13023_2016_446_MOESM3_ESM.bmp]
